# Late Delayed Bleeding After Gastric ESD with Esomeprazole/Sodium Bicarbonate Versus Conventional Esomeprazole, and Artificial Ulcer Healing with Esomeprazole/Sodium Bicarbonate: A Retrospective Cohort Study

**DOI:** 10.3390/jcm15218454

**Published:** 2026-10-30

**Authors:** Hyeong-Seok Nam, Dae-Gon Ryu, Su-Jin Kim, Su-Bum Park, Jin-Ook Jang, Woo-Jin Kim, Cheol-Min Lee, Eun-Jung Choi, Cheol-Woong Choi

**Affiliations:** 1 Department of Internal Medicine, Dong-Eui Medical Center, Busan 47227, Republic of Korea; skyace27@hanmail.net; 2 Department of Internal Medicine, Pusan National University School of Medicine and Research Institute for Convergence of Biomedical Science and Technology, Pusan National University Yangsan Hospital, Yangsan 50612, Republic of Korea; gon22gon@naver.com (D.-G.R.); pmcac@hanmail.net (S.-J.K.); psubumi@hanmail.net (S.-B.P.); a_warm_mind@naver.com (J.-O.J.); pangolinlego@naver.com (W.-J.K.); oyatrhee@gmail.com (C.-M.L.);

**Keywords:** gastric endoscopic submucosal dissection, artificial ulcer healing, delayed bleeding, esomeprazole/sodium bicarbonate, antithrombotic therapy, risk factors

## Abstract

**Background/Objectives:** Artificial ulcers after gastric endoscopic submucosal dissection (ESD) generally heal within 8 weeks with proton pump inhibitor (PPI) therapy; however, delayed bleeding remains a clinically relevant complication. Esomeprazole/sodium bicarbonate is an immediate-release PPI formulation; its role after ESD is unclear. **Methods:** This single-center retrospective study included 923 patients who underwent gastric ESD from July 2017 through December 2025. Late delayed bleeding, defined as bleeding after second-look endoscopy, was compared between patients receiving esomeprazole/sodium bicarbonate (*n* = 532) and conventional esomeprazole (*n* = 391) using Firth logistic regression adjusted for calendar period and clinical factors. Eight-week artificial ulcer healing was assessed descriptively only in the esomeprazole/sodium bicarbonate group (*n* = 527). **Results:** Late delayed bleeding occurred in 26/923 patients (2.8%): 16/532 (3.0%) in the esomeprazole/sodium bicarbonate group and 10/391 (2.6%) in the conventional esomeprazole group. PPI formulation was not associated with late delayed bleeding (adjusted OR 0.872; 95% CI 0.313–2.427). Submucosal fibrosis (adjusted OR 4.502; 95% CI 1.969–10.292), antiplatelet use (adjusted OR 3.620; 95% CI 1.617–8.103), and anticoagulant use (adjusted OR 6.783; 95% CI 1.323–34.770) were independently associated with bleeding. All 26 patients with bleeding were men; sex was therefore excluded from the model. Complete healing was observed in 510/527 patients (96.8%); ulcer diameter ≥ 40 mm was independently associated with incomplete healing (adjusted OR 7.114; 95% CI 2.664–18.998). **Conclusions:** No statistically significant difference in late delayed bleeding was detected between formulations, although a clinically meaningful difference cannot be excluded. Healing was assessed only in the esomeprazole/sodium bicarbonate group and could not be compared between formulations. Late delayed bleeding was associated with submucosal fibrosis and antithrombotic therapy.

## 1. Introduction

Gastric endoscopic submucosal dissection (ESD) has become a standard, minimally invasive treatment for early gastric neoplasms, enabling high en bloc resection rates while avoiding surgical gastrectomy in many patients. However, ESD inevitably creates a large iatrogenic mucosal defect, resulting in an artificial ulcer. Although these artificial ulcers are typically larger than conventional peptic ulcers, they follow a characteristic healing course. Histopathologic and endoscopic observations demonstrate that early contraction of the ulcer base is followed by granulation tissue formation and re-epithelialization, with most lesions progressing to the scar stage within approximately 8 weeks with adequate acid suppression [1,2]. Accordingly, an 8-week proton pump inhibitor (PPI) regimen has been widely adopted in routine post-ESD care [3,4].

Esomeprazole/sodium bicarbonate is an immediate-release PPI formulation designed for more rapid acid suppression than conventional delayed-release formulations. Conventional enteric-coated PPIs require intestinal dissolution and are subject to variable gastric emptying. In the immediate-release formulation, sodium bicarbonate neutralizes intragastric acid. This protects esomeprazole from acid degradation and facilitates absorption, shortening the time to peak plasma concentration [5]. Although 24-h acid suppression is comparable between the two formulations, earlier absorption may improve intragastric pH control during the critical early post-ESD period, when the artificial ulcer remains most vulnerable to acid-related injury and bleeding. Nevertheless, direct evidence comparing immediate-release and conventional delayed-release PPI formulations for post-ESD artificial ulcer healing and the prevention of delayed bleeding remains limited.

Despite favorable overall healing rates under PPI therapy, delayed bleeding remains one of the most clinically significant adverse events following gastric ESD, frequently necessitating urgent endoscopic intervention. A large-scale multicenter survey reported an overall postoperative bleeding rate of approximately 5.5% alongside substantial inter-institutional variability in post-ESD management strategies, indicating that an optimal preventive protocol has not been established [6]. Previous studies and pooled analyses consistently indicate that post-ESD delayed bleeding is multifactorial, driven by antithrombotic exposure as well as lesion- and procedure-related factors [7,8]. Although risk stratification tools such as the BEST-J score have been introduced to guide risk-adapted clinical management [8], no single approach fully predicts or prevents delayed bleeding, highlighting an unmet need for optimized post-ESD management strategies.

This study aimed to compare the incidence of late delayed bleeding between patients receiving immediate-release esomeprazole/sodium bicarbonate and those receiving conventional esomeprazole within a real-world cohort, and to identify independent risk factors associated with late delayed bleeding. We also described artificial ulcer healing at 8 weeks among patients treated with the esomeprazole/sodium bicarbonate formulation.

## 2. Materials and Methods

### 2.1. Study Design and Patients

This single-center retrospective study was conducted at a tertiary referral hospital and included patients who underwent gastric endoscopic submucosal dissection (ESD) between July 2017 and December 2025. During the study period, 938 gastric ESD procedures were performed for gastric adenoma, early gastric cancer, neuroendocrine tumors, or other relevant gastric neoplasms.

**Figure 1:**
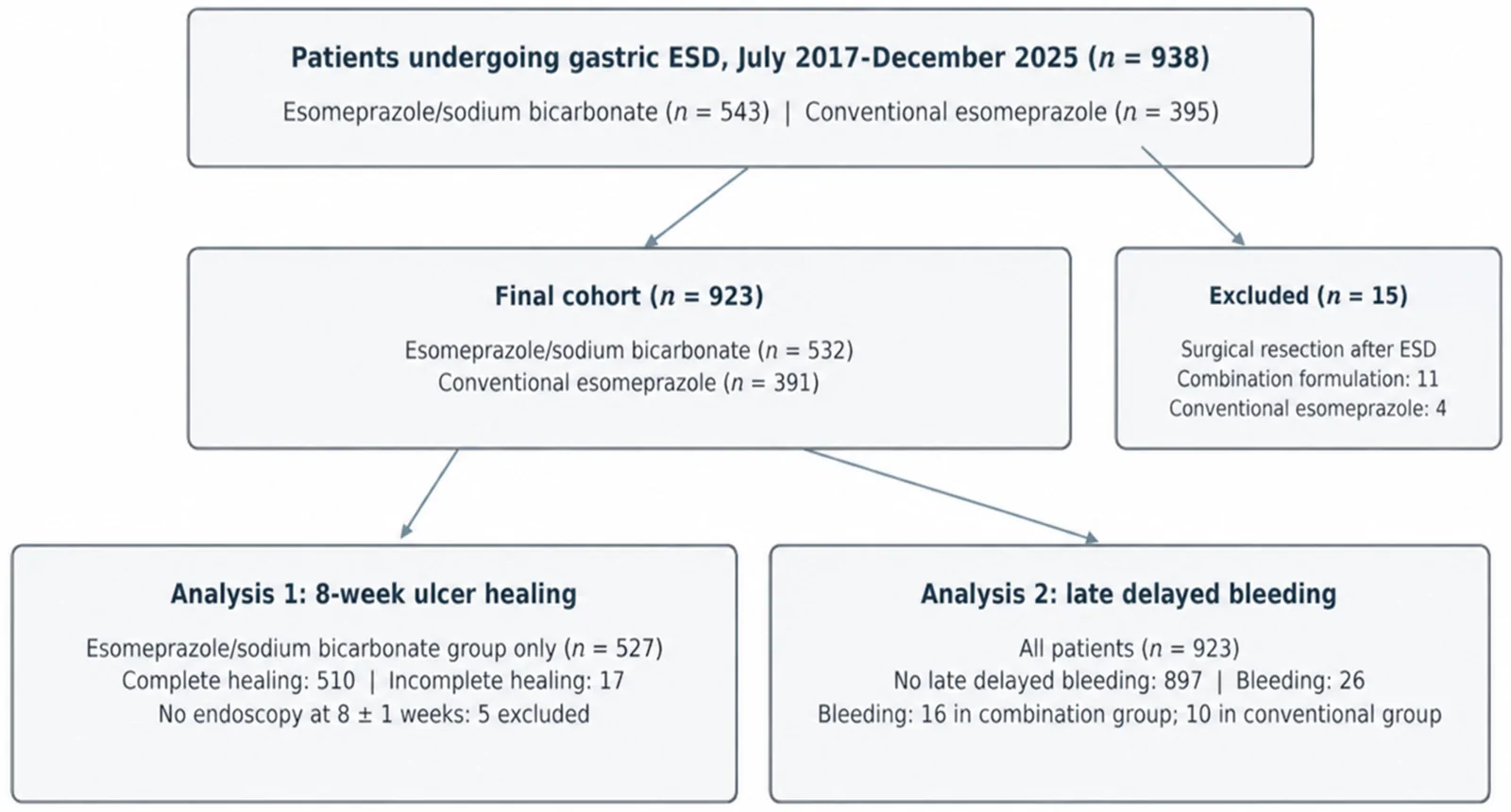
Flow diagram of patient selection and study analyses. A total of 938 patients who underwent gastric endoscopic submucosal dissection (ESD) were initially screened (esomeprazole/sodium bicarbonate group, *n* = 543; conventional esomeprazole group, *n* = 395). After excluding 15 patients who subsequently underwent surgical resection (*n* = 11 and *n* = 4, respectively), 923 patients were included in the final analysis. Analysis 1 evaluated 8-week artificial ulcer healing in 527 patients from the esomeprazole/sodium bicarbonate group who underwent follow-up endoscopy at 8 ± 1 weeks (5 patients were excluded due to missing follow-up). Analysis 2 compared late delayed bleeding between the treatment groups in the overall final cohort (*n* = 923).

Patients who subsequently required surgical resection following ESD (*n* = 15) were excluded from the analysis. After applying this exclusion criterion, the final study cohort comprised 923 patients, who were categorized into the esomeprazole/sodium bicarbonate group (*n* = 532) and the conventional esomeprazole group (*n* = 391). A patient flow diagram illustrating inclusion and exclusion is presented in Figure 1.

Analysis 1 assessed 8-week artificial ulcer healing in patients receiving esomeprazole/sodium bicarbonate who underwent follow-up endoscopy 8 ± 1 weeks after ESD (*n* = 527). Healing could not be compared between groups because only 5 patients in the conventional esomeprazole group underwent endoscopy within this time window. Follow-up endoscopy was originally scheduled at 6 months post-ESD; however, given that synchronous lesions are identified in 12.1% of post-ESD patients at our institution—with approximately one-third having been overlooked during the initial ESD [9]—the schedule was revised to 2 months post-ESD to confirm ulcer healing and detect synchronous tumors earlier. This institutional protocol modification coincided with the introduction of the esomeprazole/sodium bicarbonate formulation. Analysis 2 compared late delayed bleeding between the two treatment groups in the overall cohort (*n* = 923).

All ESD procedures were performed by a single experienced endoscopist (Cheol Woong Choi), ensuring procedural consistency and minimizing operator-related variability. This study was conducted in accordance with the ethical principles of the Declaration of Helsinki and was approved by the Institutional Review Board of Pusan National University Yangsan Hospital (IRB No. 55-2025-140). The requirement for informed consent was waived owing to the retrospective design of the study.

### 2.2. Endoscopic Submucosal Dissection Procedure

ESD was performed using a high-definition gastroscope (GIF-H290; Olympus, Tokyo, Japan) or a multibending endoscope (GIF-2TQ260M; Olympus, Tokyo, Japan), selected according to lesion location and endoscopic accessibility. An insulated-tip knife (ClearCut knife H type; Finemedix, Daegu, Republic of Korea) was used as the standard knife in all patients. A dual knife (Olympus, Tokyo, Japan) was added when needed, particularly in cases with marked submucosal fibrosis, which typically increases procedural difficulty and dissection time.

The ESD procedure followed a standardized sequence: circumferential marking around the lesion using spray coagulation mode, mucosal incision along the marking line, and submucosal dissection with repeated submucosal injections as needed. During submucosal dissection, visible vessels were prophylactically coagulated to minimize the risk of delayed bleeding. Routine second-look endoscopy was performed on postoperative day 1, and endoscopic hemostasis was performed only when high-risk stigmata—such as exposed vessels, adherent clots, oozing, or active bleeding—were identified. Procedure time was defined as the interval from the initiation of circumferential marking to the completion of coagulation of exposed vessels after resection. Artificial ulcer size was determined by measuring the maximal diameter of the resected specimen, which was pinned flat on a board immediately after ESD and measured using a standard ruler. Endoscopic lesion size was also recorded.

### 2.3. Periprocedural Management and Acid Suppression Therapy

All patients fasted until second-look endoscopy on postoperative day 1 and received intravenous esomeprazole 40 mg twice daily on the day of ESD and on postoperative day 1. In the absence of bleeding or other complications, oral acid suppression therapy was initiated on postoperative day 2.

Patients received either an immediate-release formulation of esomeprazole/sodium bicarbonate (consisting of 40 mg esomeprazole combined with 800 mg or 700 mg sodium bicarbonate once daily; ESODUO^®^/ESODUOS^®^ tablets, Chong Kun Dang Pharmaceutical Corp., Seoul, Republic of Korea) or a conventional delayed-release formulation of esomeprazole 40 mg once daily; both were taken before breakfast. The 800 mg formulation was used from February 2021, whereas the 700 mg formulation, launched in May 2023, was introduced into clinical practice in March 2025. Both formulations contain identical amounts of esomeprazole, differing only in sodium bicarbonate content, and their pharmacokinetic and pharmacodynamic comparability was established in phase 1 studies submitted for regulatory approval [10]. Oral PPI therapy was continued for 8 weeks. Treatment regimens were not individualized based on patient characteristics: conventional esomeprazole served as the standard institutional regimen until esomeprazole/sodium bicarbonate became available in February 2021, after which the protocol changed to esomeprazole/sodium bicarbonate. Following this transition, conventional esomeprazole was administered to 54 patients, mainly because of use before ESD or patient preference.

Antiplatelet agents, including aspirin, were discontinued 5 days before ESD, and direct oral anticoagulants were withheld for 1–2 days depending on renal function. Both classes of agents were resumed on postoperative day 2 if second-look endoscopy revealed no evidence of bleeding. Heparin bridging was not performed, and no patients were receiving warfarin. Helicobacter pylori infection was diagnosed based on a positive rapid urease test or histological examination prior to ESD. Infected patients received eradication therapy following the 2-month follow-up endoscopy, and eradication success was confirmed via a urea breath test 6–8 weeks later. The overall perioperative protocol—including intravenous PPI administration until second-look endoscopy, antithrombotic management, and discharge criteria—remained consistent throughout the study period.

### 2.4. Lesion Characteristics and Outcome Definitions

Lesion characteristics, including ulceration, erosion, submucosal fibrosis, a nodular surface, and mucosal redness, were assessed using diagnostic and intraoperative endoscopy. Submucosal fibrosis was defined as clearly visible fibrotic strands observed during dissection or insufficient mucosal elevation following submucosal injection. At follow-up endoscopy performed 8 ± 1 weeks after ESD, complete artificial ulcer healing was defined as full epithelial coverage with no residual ulcer bed. Only patients who underwent endoscopy within this specified window were included in the healing analysis, and the persistence of any mucosal defect was classified as incomplete healing.

Bleeding events were classified as either early bleeding—defined as bleeding occurring during ESD or prior to second-look endoscopy on postoperative day 1—or late delayed bleeding, defined as bleeding occurring after second-look endoscopy during oral PPI therapy. Late delayed bleeding was diagnosed when clinical manifestations such as hematemesis or melena necessitated endoscopic hemostasis or blood transfusion, or when a decrease in hemoglobin level of ≥2 g/dL was documented. All bleeding events were confirmed and, when indicated, managed endoscopically.

### 2.5. Outcome Measures

The primary outcome of Analysis 1 was the proportion of patients achieving complete artificial ulcer healing at 8 weeks post-ESD in the esomeprazole/sodium bicarbonate group. The primary outcome of Analysis 2 was the incidence of late delayed bleeding, compared between the esomeprazole/sodium bicarbonate and conventional esomeprazole groups. Secondary outcomes included factors associated with incomplete artificial ulcer healing (Analysis 1) and those associated with late delayed bleeding (Analysis 2). Acid-suppression-related adverse events were identified from medical records.

### 2.6. Statistical Analysis

Continuous variables were nonnormally distributed (Shapiro–Wilk test) and were compared using the Mann–Whitney U test; categorical variables were compared using the chi-square test or Fisher’s exact test, as appropriate. Because few events occurred, we used Firth penalized logistic regression with covariates prespecified on clinical grounds. The late delayed bleeding model included PPI formulation, calendar period, antiplatelet use, anticoagulant use, age and procedure time (both continuous), and submucosal fibrosis. Calendar period was dichotomized at the median procedure date (13 December 2021). Male sex was excluded because of quasi-complete separation. The incomplete healing model included age ≥65 years, ulcer diameter ≥ 40 mm, depressed morphology, and second-look endoscopic hemostasis. The age and ulcer size cutoffs were based on the conventional definition of older adults and a previous study [11]; the 10-min procedure-time cutoff corresponded to the median and was not prespecified. Sensitivity analyses assessed the robustness of the findings. No covariate data were missing. The study followed STROBE guidelines. All tests were two-sided; *p* < 0.05 was considered statistically significant. Analyses were performed in Python 3.11 with SciPy 1.17 and stats models 0.15; Firth’s penalized logistic regression was implemented in Python.

## 3. Results

### 3.1. Baseline Characteristics

The final analysis included 923 patients: 532 in the esomeprazole/sodium bicarbonate group and 391 in the conventional esomeprazole group (Table 1).

Patients receiving esomeprazole/sodium bicarbonate were older (median age, 68 vs. 62 years; *p* < 0.001) and had shorter procedure times (median, 10 vs. 13 min; *p* < 0.001); most procedures in this group occurred later in the study period. The proportion of men was similar between groups (67.1% vs. 72.6%; *p* = 0.072). Artificial ulcer diameter was slightly smaller in the combination group (median, 28 vs. 30 mm; *p* = 0.010), whereas lesion size was similar (median, 14 mm in both groups; *p* = 0.398).

Groups did not differ significantly in depressed morphology, submucosal fibrosis, en bloc resection, second-look endoscopic hemostasis, submucosal invasion, antiplatelet use, anticoagulant use, diabetes, or hypertension (all *p* > 0.05).

### 3.2. Artificial Ulcer Healing at 8 Weeks (Esomeprazole/Sodium Bicarbonate Group)

Among 527 patients receiving esomeprazole/sodium bicarbonate who underwent endoscopy 8 ± 1 weeks after ESD, 510 (96.8%) had complete healing and 17 (3.2%) had incomplete healing (Table 2).

On univariable analysis, age ≥ 65 years, ulcer diameter ≥ 40 mm, depressed morphology, and second-look endoscopic hemostasis were associated with incomplete healing (all *p* < 0.05).

On multivariable analysis, ulcer diameter ≥ 40 mm (adjusted OR 7.114; 95% CI 2.664–18.998; *p* < 0.001) and depressed morphology (adjusted OR 2.706; 95% CI 1.004–7.292; *p* = 0.049) were independently associated with incomplete healing (Appendix A Table A1). Age ≥65 years showed a borderline association (adjusted OR 3.683; 95% CI 0.947–14.322; *p* = 0.060). When age and ulcer size were modeled continuously, each 10-mm increase in ulcer size was associated with an OR of 2.026 (95% CI 1.475–2.783).

### 3.3. Late Delayed Bleeding

Late delayed bleeding occurred in 26/923 patients (2.8%). Early bleeding occurred in 16 patients (1.7%); these patients remained in the cohort but were not counted as late delayed bleeding events. The incidence was 3.0% (16/532) with esomeprazole/sodium bicarbonate and 2.6% (10/391) with conventional esomeprazole (*p* = 0.683; Table 3).

On univariable analysis, submucosal fibrosis, antiplatelet use, anticoagulant use, longer procedure time, and second-look endoscopic hemostasis were associated with late delayed bleeding. All patients with bleeding were men; sex was therefore excluded from the multivariable model.

After adjustment, PPI formulation was not associated with late delayed bleeding (adjusted OR 0.872; 95% CI 0.313–2.427; *p* = 0.793). Submucosal fibrosis (adjusted OR 4.502; 95% CI 1.969–10.292; *p* < 0.001), antiplatelet use (adjusted OR 3.620; 95% CI 1.617–8.103; *p* = 0.002), and anticoagulant use (adjusted OR 6.783; 95% CI 1.323–34.770; *p* = 0.022) were independently associated with bleeding (Appendix A Table A2). Calendar period was not associated with bleeding (adjusted OR 1.478; 95% CI 0.548–3.986). In an additional model, second-look endoscopic hemostasis was also associated with bleeding (adjusted OR 2.942; 95% CI 1.318–6.564; *p* = 0.008; Appendix A Table A3).

**Figure A1:**
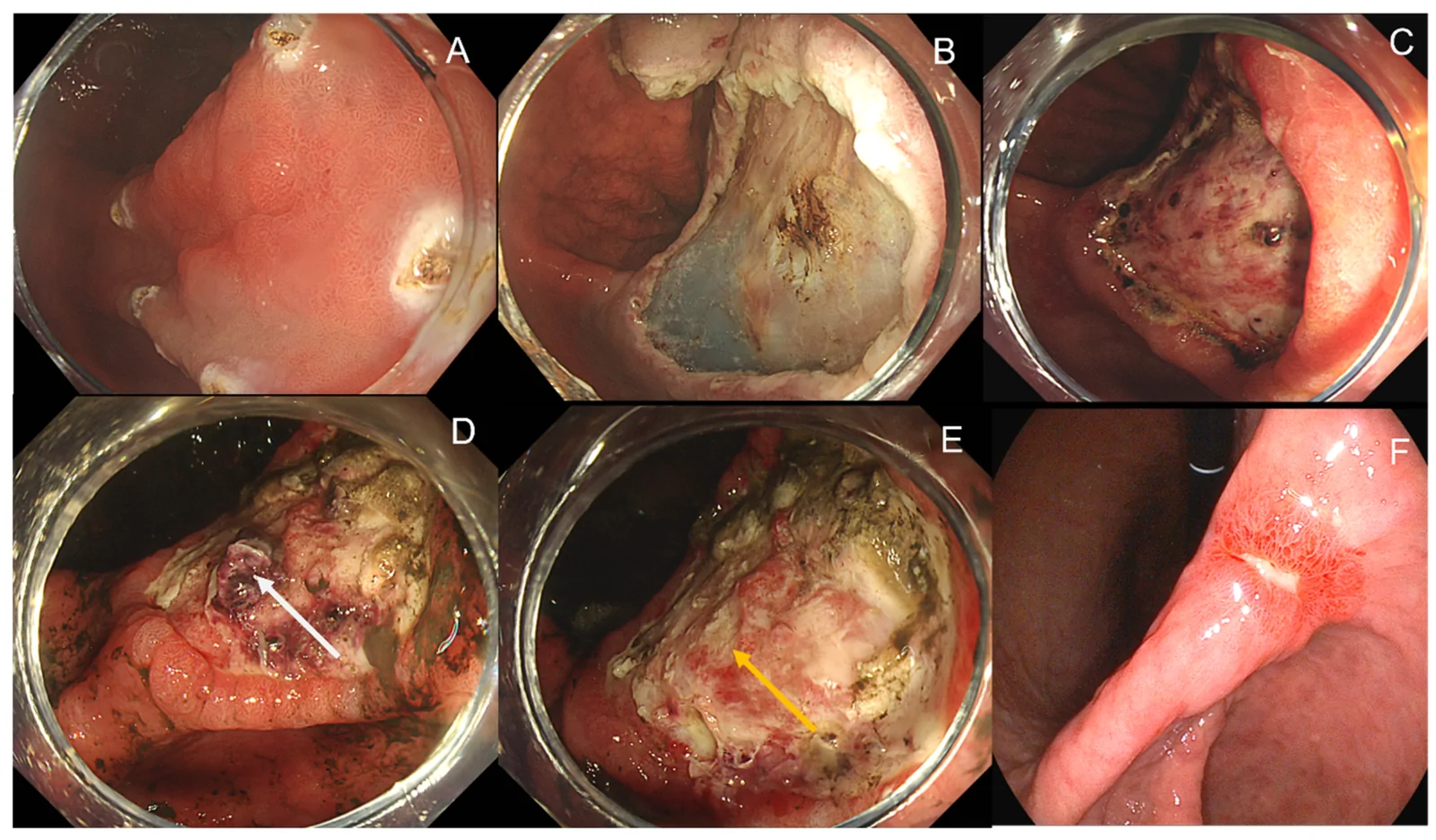
Clinical course of delayed bleeding and incomplete healing of an artificial ulcer after gastric ESD in a patient receiving antiplatelet therapy in the esomeprazole/sodium bicarbonate group. (**A**) Lesion after circumferential marking and before ESD. (**B**) Artificial ulcer immediately after ESD. (**C**) Second-look endoscopy on post-ESD day 1, showing no active bleeding. Clopidogrel was resumed on post-ESD day 2. (**D**) Endoscopy performed 10 days after ESD for hematemesis, showing a large artificial ulcer with an adherent clot at the suspected bleeding site (white arrow). (**E**) Endoscopic appearance after coagulation therapy for hemostasis of the suspected bleeding site (yellow arrow). (**F**) Follow-up endoscopy at 2 months after ESD showing incomplete healing of the artificial ulcer.

Results were similar when ulcer size or lesion location was added to the model, when age and procedure time were dichotomized, and when analyses were restricted to men (Appendix A Table A3). Estimates were also similar across calendar periods and subgroups (Appendix A Table A4 and Table A5). Appendix B Figure A1 shows a representative case of delayed bleeding and incomplete ulcer healing in a patient receiving antiplatelet therapy.

## 4. Discussion

In this retrospective cohort of 923 patients undergoing gastric ESD, late delayed bleeding occurred in 3.0% (16/532) of patients in the esomeprazole/sodium bicarbonate group and 2.6% (10/391) in the conventional esomeprazole group. The PPI formulation was not significantly associated with late delayed bleeding after adjustment for calendar period and clinical factors (adjusted OR, 0.872; 95% CI, 0.313–2.427). However, a clinically meaningful difference could not be excluded, and artificial ulcer healing could not be compared between groups. Among patients receiving esomeprazole/sodium bicarbonate, complete healing at 8 weeks was observed in 96.8% (510/527). Overall, late delayed bleeding was primarily associated with lesion-related and patient-related factors rather than with the specific PPI formulation.

Despite its pharmacokinetic advantages, esomeprazole/sodium bicarbonate was not associated with a lower rate of late delayed bleeding compared with conventional esomeprazole—an outcome that was not entirely unexpected. All patients received intravenous esomeprazole until second-look endoscopy on postoperative day 1 before transitioning to oral therapy, which may have attenuated any differential effect of the oral formulation on early bleeding risk. In clinical practice, the primary rationale for selecting esomeprazole/sodium bicarbonate was its potential for more rapid relief of postprocedural epigastric discomfort during the active healing phase, rather than an expectation of superior ulcer healing or bleeding prevention. However, symptom-related outcomes were not systematically documented in medical records owing to the retrospective study design and could not be compared between groups, warranting evaluation in prospective studies.

The two treatment groups differed in age and procedure time. Because the routine formulation changed over time, these differences largely reflect the calendar period: procedure time decreased over time in both groups, most likely reflecting the increasing experience of a single endoscopist, while the treated population became older. Calendar period, age, and procedure time were therefore included in the multivariable model. Although the artificial ulcer diameter was slightly smaller in the esomeprazole/sodium bicarbonate group, the difference was minimal, and incorporating ulcer size into the model did not alter the results (Appendix A Table A3). In an exploratory analysis, late delayed bleeding was more frequent with the 700 mg than with the 800 mg sodium bicarbonate formulation (5.8% vs. 1.5%, *p* = 0.005). However, the 700 mg formulation was used only in 2025, a period during which antiplatelet use and second-look endoscopic hemostasis were also more frequent in the conventional esomeprazole group. After adjustment for second-look endoscopic hemostasis, this difference was no longer significant, and within 2025, late delayed bleeding did not differ significantly between the 700 mg formulation and conventional esomeprazole (Appendix A Table A6). Because formulation and period effects could not be completely separated and the number of events was small, these data neither establish nor exclude a difference between the two formulations, which warrants evaluation in future prospective studies.

The favorable healing outcomes observed in our cohort can be better understood by considering the distinct biological characteristics of artificial ulcers created by gastric ESD. Unlike chronic peptic ulcers, which often arise against a background of sustained acid injury and long-standing inflammation with established submucosal fibrosis, ESD-induced ulcers are acute iatrogenic mucosal defects created in otherwise viable gastric wall structures. Histopathologic analyses have demonstrated that the early healing phase is characterized by rapid contraction of the ulcer base, followed by organized granulation tissue formation and progressive re-epithelialization [1]. Even in large defects, contraction of the ulcer base supported by the intact underlying muscle layer contributes to structural stabilization, allowing most lesions to reach the scar stage within approximately 8 weeks under PPI therapy [1,3,4].

In this context, complete healing was observed in 96.8% of patients receiving esomeprazole/sodium bicarbonate. Because healing could not be directly compared with the conventional esomeprazole group, this finding remains descriptive and does not establish comparable healing efficacy between formulations. Incomplete healing was independently associated with artificial ulcer diameter ≥ 40 mm (adjusted OR, 7.114; 95% CI, 2.664–18.998) and depressed morphology (adjusted OR, 2.706; 95% CI, 1.004–7.292), while older age showed a borderline association, suggesting that defect burden and host regenerative capacity may limit artificial ulcer repair. Larger resection size directly reflects a greater mucosal and submucosal defect, requiring a broader area of contraction, granulation, and re-epithelialization. Although artificial ulcers typically undergo rapid contraction, extensive defects may exceed the mechanical and biological capacity for complete remodeling within the standard 8-week period. Similarly, advanced age is associated with impaired mucosal regeneration, reduced angiogenic response, and delayed epithelial restitution, all of which may impede timely ulcer closure despite adequate acid suppression. Depressed morphology may reflect ulceration or deeper submucosal involvement of the lesion, which could delay re-epithelialization; however, this association was only marginally statistically significant and should be interpreted cautiously. These findings are consistent with prior studies reporting larger specimen size and older age as independent risk factors for delayed artificial ulcer healing after gastric ESD [12,13].

Late delayed bleeding in our cohort was associated with lesion complexity and antithrombotic therapy rather than with differences in the acid suppression strategy. Submucosal fibrosis likely reflects technically demanding dissection, in which dense fibrotic tissue requires more extensive submucosal manipulation and thermal coagulation, potentially increasing the likelihood of residual or inadequately sealed vessels within the artificial ulcer base [6,7,8]. Although these mechanisms are plausible explanations for the observed association, they could not be directly tested in this study. Longer procedure time was associated with late delayed bleeding in the univariable analysis but not after multivariable adjustment, suggesting that this association may be partly explained by submucosal fibrosis, which can prolong dissection time. Antithrombotic therapy may further exacerbate the intrinsic vulnerability of the post-ESD ulcer base. Even after apparently successful endoscopic hemostasis, the large exposed vascular surface created by ESD remains susceptible to rebleeding under platelet inhibition or anticoagulation, particularly during the early reparative phase before stable re-epithelialization has been established [6,7,8]. The association between second-look endoscopic hemostasis and delayed bleeding most likely reflects the underlying severity of the ulcer rather than a direct adverse effect of the intervention itself. Because second-look endoscopic hemostasis was performed exclusively when high-risk stigmata were present, it identified ulcers that were already at high risk of bleeding; this association may therefore reflect confounding by indication. This interpretation is consistent with prior large-scale analyses and risk prediction models, including the BEST-J score, which emphasize lesion-related factors and antithrombotic exposure as principal determinants of post-ESD bleeding risk [6,7,8]. As illustrated in Appendix B Figure A1, delayed bleeding in patients receiving antiplatelet therapy may also be associated with prolonged artificial ulcer healing, even under adequate acid suppression. All patients who experienced late delayed bleeding were men. Male sex has previously been identified as a risk factor for bleeding after gastric ESD [14], and male patients in our cohort more frequently exhibited submucosal fibrosis; unmeasured behavioral factors such as smoking, alcohol consumption, and physical activity may also contribute, though these data were unavailable.

Despite continuous efforts to reduce post-ESD delayed bleeding, a universally effective preventive strategy has not yet been established. Technical refinements during ESD, such as the aspiration method aimed at improving visualization and minimizing residual blood pooling within the artificial ulcer base, have been associated with reduced bleeding rates in selected cohorts [15,16]. Furthermore, mechanical shielding techniques using materials such as polyglycolic acid sheets or polyethylene oxide adhesives have been evaluated to protect the ulcer surface from mechanical trauma and chemical irritation, with propensity score-matched analyses suggesting potential benefits in high-risk patients [17]. Pharmacologic adjuncts, including combination regimens incorporating mucosal protective agents such as rebamipide or sequential ecabet sodium therapy in addition to PPIs, have been associated with improved ulcer healing; however, randomized controlled trials have not consistently demonstrated definitive reductions in delayed bleeding incidence [18,19]. Even with these procedural and pharmacologic refinements, bleeding risk remains closely tied to intrinsic lesion complexity and antithrombotic exposure—findings that are consistent with our multivariable analysis. Collectively, these observations suggest that delayed bleeding after gastric ESD is unlikely to be eliminated by a single intervention; rather, it reflects the complex interaction between vascular vulnerability within a large artificial ulcer and patient-related hemostatic factors. Accordingly, preventive strategies should prioritize the identification of high-risk patients—particularly those with severe submucosal fibrosis or those receiving antithrombotic therapy—and consider intensified surveillance or tailored periprocedural management rather than relying solely on the modification of acid suppression.

This study has several strengths. All ESD procedures were performed by a single experienced endoscopist, minimizing operator-related variability and ensuring procedural consistency throughout the study period. Additionally, the relatively large sample size of 923 patients from a real-world clinical setting allowed comparison of late delayed bleeding between formulations and the identification of associated risk factors through multivariable analysis. To our knowledge, this is among the first studies to evaluate the real-world clinical outcomes of esomeprazole/sodium bicarbonate in the setting of post-ESD management, encompassing both artificial ulcer healing and delayed bleeding.

Nevertheless, several limitations should be acknowledged. First, the retrospective, single-center design inherently limits the generalizability of our findings. Because the routine PPI regimen changed over time, treatment allocation was strongly associated with the calendar period, making the comparison predominantly a before-and-after analysis. Although calendar period, age, and procedure time were included in the multivariable model, the calendar period was dichotomized at the median procedure date (13 December 2021), which did not correspond to the actual transition to esomeprazole/sodium bicarbonate in February 2021. This binary cutoff may fail to adequately capture secular or gradual changes in operator experience, case mix, endoscopic practice, or other time-dependent variables. Although procedural and periprocedural protocols remained unchanged, residual temporal confounding cannot be entirely excluded. Exploratory analyses stratified by calendar period yielded imprecise estimates owing to the small number of bleeding events. Therefore, these findings should be interpreted strictly as observational associations rather than as evidence of equivalence or a causal treatment effect. Second, 8-week follow-up endoscopy was performed in only 5 patients in the conventional esomeprazole group because of an institutional protocol change; consequently, healing could not be formally compared between formulations and was evaluated exclusively within the esomeprazole/sodium bicarbonate group. Third, only 26 late delayed bleeding events occurred, and the wide confidence intervals—particularly for anticoagulant use (adjusted OR, 6.783; 95% CI, 1.323–34.770)—indicate that a clinically meaningful difference could not be excluded between formulations. Furthermore, all patients who experienced late delayed bleeding were men, limiting the generalizability of the bleeding model to female patients. Fourth, the 700 mg sodium bicarbonate formulation was used exclusively in 2025, preventing the clear separation of formulation effects from period effects (Appendix A Table A6). Fifth, all patients routinely underwent second-look endoscopy under institutional policy. This practice is not recommended for unselected patients in current guidelines and may limit generalizability; endoscopic hemostasis performed when indicated at this examination may also have lowered the observed bleeding rate. Sixth, medication adherence, CYP2C19 genotype, prior Helicobacter pylori eradication status, smoking history, and alcohol consumption could not be assessed [12]. Finally, this study was supported by a research grant from the manufacturer of esomeprazole/sodium bicarbonate, representing a potential source of bias, although the funder had no role in the design, conduct, data analysis, or reporting of the study.

## 5. Conclusions

In this retrospective cohort, late delayed bleeding did not differ significantly between esomeprazole/sodium bicarbonate and conventional esomeprazole after gastric ESD, although a clinically meaningful difference cannot be excluded. Eight-week artificial ulcer healing was high in the combination group but was not compared between formulations. Late delayed bleeding was associated with submucosal fibrosis and antithrombotic therapy.

## Figures and Tables

**Table 1:** Baseline characteristics of patients receiving esomeprazole/sodium bicarbonate or conventional esomeprazole after gastric ESD.

Characteristics	Esomeprazole/Sodium Bicarbonate (*n* = 532)	Esomeprazole (*n* = 391)	Total (*n* = 923)	*p* Value
Male, *n* (%)	357 (67.1)	284 (72.6)	641 (69.4)	0.072
Age, years, median (IQR)	68 (62–73)	62 (54–68)	66 (59–72)	<0.001
Age ≥ 65 years, *n* (%)	347 (65.2)	151 (38.6)	498 (54.0)	<0.001
Endoscopic lesion size, mm, median (IQR)	14 (10–20)	14 (10–19)	14 (10–19)	0.398
Artificial ulcer diameter, mm, median (IQR)	28 (21–35)	30 (25–35)	30 (22–35)	0.010
Procedure time, min, median (IQR)	10 (8–15)	13 (9–20)	10 (8–17)	<0.001
Depressed morphology, *n* (%)	217 (40.8)	165 (42.2)	382 (41.4)	0.667
Submucosal fibrosis, *n* (%)	75 (14.1)	53 (13.6)	128 (13.9)	0.814
En-bloc resection, *n* (%)	522 (98.1)	388 (99.2)	910 (98.6)	0.156
Second-look endoscopic hemostasis, *n* (%)	133 (25.0)	114 (29.2)	247 (26.8)	0.159
Submucosal invasion, *n* (%)	20 (3.8)	11 (2.8)	31 (3.4)	0.430
Location, *n* (%)				0.906
Upper third	46 (8.6)	37 (9.5)	83 (9.0)	
Middle third	156 (29.3)	115 (29.4)	271 (29.4)	
Lower third	330 (62.0)	239 (61.1)	569 (61.6)	
Histology, *n* (%)				0.188
Adenoma	376 (70.7)	268 (68.5)	644 (69.8)	
Adenocarcinoma	153 (28.8)	116 (29.7)	269 (29.1)	
Other ^a^	3 (0.6)	7 (1.8)	10 (1.1)	
Antiplatelet agents, *n* (%)	99 (18.6)	74 (18.9)	173 (18.7)	0.903
Anticoagulants, *n* (%)	9 (1.7)	3 (0.8)	12 (1.3)	0.221
Diabetes mellitus, *n* (%)	117 (22.0)	70 (17.9)	187 (20.3)	0.127
Hypertension, *n* (%)	256 (48.1)	183 (46.8)	439 (47.6)	0.692
Helicobacter pylori infection, *n* (%) ^b^	185 (34.8)	150 (38.4)	335 (36.3)	0.263
Procedure after 13 December 2021, *n* (%)	410 (77.1)	51 (13.0)	461 (49.9)	<0.001

Continuous variables are presented as medians (IQRs) and compared using the Mann–Whitney U test; categorical variables are presented as *n* (%) and compared using the chi-square test or Fisher’s exact test. ^a^ Neuroendocrine tumor (*n* = 8) and lymphoma (*n* = 2). ^b^ Positive rapid urease test or histology before ESD. Antiplatelet agents: aspirin (*n* = 64), clopidogrel (*n* = 46), cilostazol (*n* = 28), anagrelide (*n* = 9), sarpogrelate (*n* = 6), limaprost (*n* = 4), and dual antiplatelet therapy (*n* = 15); anticoagulants: apixaban (*n* = 11, including one patient also taking aspirin) and edoxaban (*n* = 1). IQR, interquartile range; ESD, endoscopic submucosal dissection.

**Table 2:** Artificial ulcer healing outcomes in patients receiving esomeprazole/sodium bicarbonate at 8 weeks after gastric ESD (*n* = 527): univariable and multivariable analyses.

Characteristics	Incomplete Healing (*n* = 17)	Complete Healing (*n* = 510)	*p* Value	Adjusted OR (95% CI)	*p* Value
Male, *n* (%)	12 (70.6)	341 (66.9)	0.748		
Age ≥ 65 years, *n* (%)	15 (88.2)	330 (64.7)	0.045	3.683 (0.947–14.322)	0.060
Antiplatelet agent use, *n* (%)	6 (35.3)	92 (18.0)	0.104		
Anticoagulant use, *n* (%)	0 (0.0)	9 (1.8)	1.000		
Artificial ulcer diameter ≥ 40 mm, *n* (%)	11 (64.7)	85 (16.7)	<0.001	7.114 (2.664–18.998)	<0.001
Procedure time, min, median (IQR)	10 (10–20)	10 (8–15)	0.064		
Depressed morphology, *n* (%)	11 (64.7)	203 (39.8)	0.040	2.706 (1.004–7.292)	0.049
Submucosal fibrosis, *n* (%)	5 (29.4)	68 (13.3)	0.072		
Helicobacter pylori infection, *n* (%)	7 (41.2)	176 (34.5)	0.570		
Second-look endoscopic hemostasis, *n* (%)	8 (47.1)	123 (24.1)	0.044	1.849 (0.700–4.886)	0.215

Values are presented as *n* (%) or median (IQR). Adjusted odds ratios (ORs) and 95% confidence intervals (CIs) are from Firth penalized logistic regression including age ≥ 65 years, artificial ulcer diameter ≥ 40 mm, depressed morphology, and second-look endoscopic hemostasis. Patients without endoscopic assessment at 8 ± 1 weeks (*n* = 5) were excluded. When age and artificial ulcer size were entered as continuous variables, the OR for artificial ulcer size was 2.026 per 10 mm (95% CI 1.475–2.783). Helicobacter pylori infection was defined as in Table 1. ESD, endoscopic submucosal dissection; IQR, interquartile range.

**Table 3:** Factors associated with late delayed bleeding after gastric ESD (*n* = 923): univariable and multivariable analyses.

Variables	No Late Delayed Bleeding (*n* = 897)	Late Delayed Bleeding (*n* = 26)	Univariable OR (95% CI)	*p* Value	Adjusted OR (95% CI)	*p* Value
Esomeprazole/sodium bicarbonate, *n* (%)	516 (57.5)	16 (61.5)	1.161 (0.529–2.547)	0.710	0.872 (0.313–2.427)	0.793
Procedure after 13 December 2021, *n* (%)	446 (49.7)	15 (57.7)	1.363 (0.628–2.958)	0.433	1.478 (0.548–3.986)	0.440
Male, *n* (%)	615 (68.6)	26 (100.0)	24.326 (1.470–402.572)	0.026		
Age, years, median (IQR) ^a^	65 (59–71)	71 (64–76)	1.445 (0.960–2.174)	0.078	1.217 (0.772–1.919)	0.397
Procedure time, min, median (IQR) ^b^	10 (8–16)	14 (10–20)	1.284 (1.054–1.565)	0.013	1.140 (0.897–1.449)	0.282
Submucosal fibrosis, *n* (%)	117 (13.0)	11 (42.3)	4.928 (2.239–10.849)	<0.001	4.502 (1.969–10.292)	<0.001
Antiplatelet agents, *n* (%)	162 (18.1)	11 (42.3)	3.358 (1.535–7.348)	0.002	3.620 (1.617–8.103)	0.002
Anticoagulants, *n* (%)	10 (1.1)	2 (7.7)	8.625 (1.942–38.298)	0.005	6.783 (1.323–34.770)	0.022
Second-look endoscopic hemostasis, *n* (%)	233 (26.0)	14 (53.8)	3.301 (1.525–7.145)	0.002		

Odds ratios (ORs) and 95% confidence intervals (CIs) were estimated using Firth penalized logistic regression. Covariates were prespecified on clinical grounds. Male sex was excluded because all 26 bleeding events occurred in men (quasi-complete separation). Second-look endoscopic hemostasis was considered a marker of ulcer severity and was included only in an additional model (Appendix A Table A3). ^a^, per 10 years. ^b^, per 10 min. ESD, endoscopic submucosal dissection; IQR, interquartile range.

## Data Availability

The data presented in this study are available from the corresponding author upon reasonable request, subject to ethical restrictions.

## Abbreviations

ESD	Endoscopic submucosal dissection
PPI	Proton pump inhibitor
OR	Odds ratio
CI	Confidence interval

## Appendix A

**Table A1:** Factors associated with incomplete artificial ulcer healing (esomeprazole/sodium bicarbonate group, *n* = 527; 17 events).

Variable	Adjusted OR	95% CI	*p* Value
Age ≥ 65 years	3.683	0.947–14.322	0.060
Artificial ulcer diameter ≥ 40 mm	7.114	2.664–18.998	<0.001
Depressed morphology	2.706	1.004–7.292	0.049
Second-look endoscopic hemostasis	1.849	0.700–4.886	0.215

Firth penalized logistic regression. Late delayed bleeding was excluded because it occurred before the healing assessment.

**Table A2:** Factors associated with late delayed bleeding (*n* = 923; 26 events).

Variable	Adjusted OR	95% CI	*p* Value
Esomeprazole/sodium bicarbonate	0.872	0.313–2.427	0.793
Procedure after 13 December 2021	1.478	0.548–3.986	0.440
Antiplatelet agents	3.620	1.617–8.103	0.002
Anticoagulants	6.783	1.323–34.770	0.022
Age, per 10 years	1.217	0.772–1.919	0.397
Procedure time, per 10 min	1.140	0.897–1.449	0.282
Submucosal fibrosis	4.502	1.969–10.292	<0.001

Firth penalized logistic regression. Male sex was excluded because all patients with late delayed bleeding were men.

**Table A3:** Association between esomeprazole/sodium bicarbonate and late delayed bleeding in the primary and additional models.

Model	Change from the Primary Model	*n*/Events	OR (95% CI) ^a^	*p* Value
Main model (Table 3)	—	923/26	0.872 (0.313–2.427)	0.793
Model 1	Calendar period removed	923/26	1.110 (0.483–2.553)	0.806
Model 2	Artificial ulcer size (per 10 mm) added	923/26	0.920 (0.335–2.523)	0.871
Model 3	Lesion location added	923/26	0.844 (0.313–2.280)	0.738
Model 4	Second-look endoscopic hemostasis added	923/26	1.015 (0.354–2.905)	0.978
Model 5	Age (≥ 65 years) and procedure time (> 10 min) dichotomized	923/26	0.865 (0.315–2.375)	0.778
Model 6	Men only	641/26	0.833 (0.288–2.407)	0.735

^a^ OR > 1 indicates higher odds of late delayed bleeding with esomeprazole/sodium bicarbonate than with conventional esomeprazole. All models used Firth penalized logistic regression. The primary model (Table 3) included PPI formulation, calendar period, antiplatelet use, anticoagulant use, age (per 10 years), procedure time (per 10 min), and submucosal fibrosis. Lesions were located in the lower third in 569 patients (12 bleeding events), the middle third in 271 (10 events), and the upper third in 83 (4 events). In Model 3, adjusted ORs were 1.727 (95% CI 0.747–3.992) for the middle third and 1.844 (95% CI 0.601–5.659) for the upper third (reference: lower third). In Model 4, second-look endoscopic hemostasis was associated with late delayed bleeding (adjusted OR 2.942, 95% CI 1.318–6.564; *p* = 0.008). OR, odds ratio; CI, confidence interval.

**Table A4:** Late delayed bleeding by calendar period (median date, 13 December 2021).

Period	*n* (Esomeprazole/Esomeprazole/Sodium Bicarbonate)	Late Delayed Bleeding, *n* (%)	Procedure Time, Min, Median	Adjusted OR for Esomeprazole/Sodium Bicarbonate (95% CI)
Early (July 2017–13 December 2021)	462 (340/122)	11 (2.4)	13	0.889 (0.207–3.818)
Late (15 December 2021–December 2025)	461 (51/410)	15 (3.3)	8	1.245 (0.238–6.513)

Adjusted for antiplatelet use, anticoagulant use, age, procedure time, and submucosal fibrosis (Firth logistic regression). Procedure time was shorter in the late period in both groups (esomeprazole: median, 14 vs. 9 min; esomeprazole/sodium bicarbonate: 10.5 vs. 8 min; both *p* < 0.001). Late delayed bleeding did not differ by period (late vs. early: adjusted OR 1.478; 95% CI 0.548–3.986; Table 3).

**Table A5:** Subgroup analyses of the association between PPI formulation and late delayed bleeding.

Subgroup	*n*/Events	Adjusted OR for Esomeprazole/Sodium Bicarbonate (95% CI)	*p* for Interaction
Antithrombotic use: yes	184/13	0.533 (0.133–2.142)	0.418
Antithrombotic use: no	739/13	1.504 (0.373–6.061)	
Procedure time ≤ 10 min	488/9	0.875 (0.173–4.420)	0.175
Procedure time > 10 min	435/17	0.935 (0.261–3.350)	

The primary model (Table 3) included PPI formulation, calendar period, antiplatelet use, anticoagulant use, age (per 10 years), procedure time (per 10 min), and submucosal fibrosis. Firth logistic regression models were adjusted for the covariates in the primary model, except the stratifying variable. These analyses are exploratory because few events occurred.

**Table A6:** Late delayed bleeding by esomeprazole/sodium bicarbonate formulation (40/700 mg vs. 40/800 mg).

**(A) Comparison Between the Two Formulations (*n* = 532)**			
	**40/800 mg (*n* = 343)**	**40/700 mg (*n* = 189)**	***p* Value**
Period of use	February 2021–December 2024	March 2025–December 2025	
Late delayed bleeding, *n* (%)	5 (1.5)	11 (5.8)	0.005
Antiplatelet use, *n* (%)	46 (13.4)	53 (28.0)	<0.001
Second-look endoscopic hemostasis, *n* (%)	45 (13.1)	88 (46.6)	<0.001
OR for late delayed bleeding (95% CI) ^a^			
Unadjusted	1 (reference)	3.965 (1.410–11.152)	0.009
Adjusted for antithrombotic use, age, procedure time and submucosal fibrosis	1 (reference)	4.326 (1.494–12.520)	0.007
Further adjusted for second-look endoscopic hemostasis	1 (reference)	2.257 (0.694–7.336)	0.176
**(B) Patients Treated in 2025 only: 40/700 mg Formulation Versus Conventional Esomeprazole (*n* = 229)**			
	**Conventional Esomeprazole (*n* = 40)**	**40/700 mg (*n* = 189)**	***p* Value**
Late delayed bleeding, *n* (%)	1 (2.5)	11 (5.8)	0.697
Antiplatelet use, *n* (%)	9 (22.5)	53 (28.0)	0.474
Second-look endoscopic hemostasis, *n* (%)	17 (42.5)	88 (46.6)	0.640
OR for late delayed bleeding, adjusted for antiplatelet use (95% CI) ^a^	1 (reference)	1.577 (0.273–9.113)	0.611

^a^ OR > 1 indicates higher odds of late delayed bleeding in the column group than in the reference group (Firth penalized logistic regression). Proportions were compared using the chi-square test or Fisher’s exact test. In (A), the difference between formulations was not significant after adjustment for second-look endoscopic hemostasis. In (B), bleeding and second-look endoscopic hemostasis did not differ between the 700 mg formulation and conventional esomeprazole within 2025. Because the 700 mg formulation was used only in 2025, formulation and period effects could not be separated. OR, odds ratio; CI, confidence interval.
